# A coleção de mosquitos de Antonio Gonçalves Peryassú do Museu Nacional, Rio de Janeiro: registro de memória de um patrimônio desaparecido

**DOI:** 10.1590/S0104-59702023000100018

**Published:** 2023-05-15

**Authors:** Ricardo Lourenço-de-Oliveira, Francisco dos Santos Lourenço

**Affiliations:** i Pesquisador, Laboratório de Mosquitos Transmissores de Hematozoários/Instituto Oswaldo Cruz/Fiocruz. Rio de Janeiro – RJ – Brasil lourenco@ioc.fiocruz.br; ii Pesquisador, Departamento de Arquivo e Documentação/Casa de Oswaldo Cruz/Fiocruz. Rio de Janeiro – RJ – Brasil francisco.lourenco@fiocruz.br

**Keywords:** Antonio Gonçalves Peryassú (1879-1962), coleção, mosquitos, Museu Nacional, Antonio Gonçalves Peryassú (1879-1962), collection, mosquitoes, Museu Nacional

## Abstract

As coleções e pesquisas feitas nas primeiras décadas do século XX, no Rio de Janeiro, foram fundamentais para o estudo da sistemática e da história natural dos mosquitos no Brasil. Um personagem de destaque nesse cenário foi Antonio Gonçalves Peryassú. Analisamos o histórico de uma coleção por ele organizada no Museu Nacional do Rio de Janeiro, entre 1918 e 1922.

## Abstract (en)

Collections formed and studies conducted in the early decades of the twentieth century in Rio de Janeiro, Brazil, were crucial for the study of the systematization and natural history of mosquitoes in Brazil. One key figure in this context was Antonio Gonçalves Peryassú. The history of a collection he organized at Museu Nacional [National Museum] in Rio de Janeiro between 1918 and 1922 is analyzed.

Antonio Gonçalves Peryassú (1879-1962) ( Figura 1 ), médico, sanitarista e professor, foi um dos pioneiros estudiosos de mosquitos no Brasil (Lourenço-de-Oliveira, Lourenço, 2022), sendo a sua tese de doutoramento na Faculdade de Medicina do Rio de Janeiro, *Os culicídeos do Brasil* , um compêndio sobre a história natural, a distribuição geográfica e a sistemática das espécies de mosquitos brasileiros ( Peryassú, 1908 ). A tese foi desenvolvida no Instituto Soroterápico Federal (Instituto de Manguinhos), rebatizado como Instituto Oswaldo Cruz em 1908, sob a orientação de Oswaldo Cruz (1872-1917) e Arthur Neiva (1880-1943).

**Figura 1:**
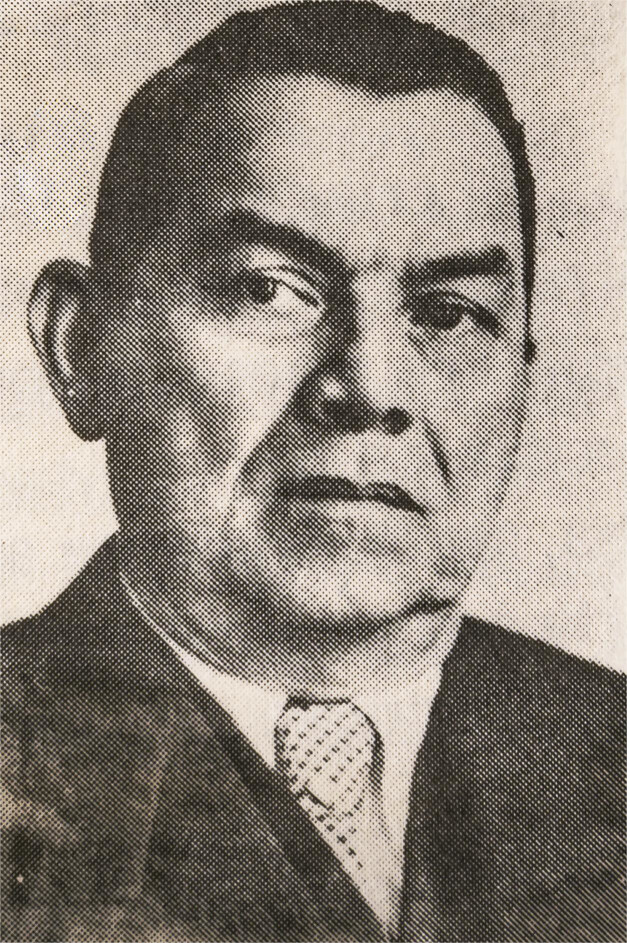
Antonio Gonçalves Peryassú ( Costa, 1973 , p.215)

A geração do conhecimento sobre a biodiversidade da fauna culicidiana registrada na tese de Peryassú resultou da coleta de espécimes não só no Rio de Janeiro, mas em várias localidades brasileiras, por ele e colaboradores, especialmente aqueles de Manguinhos, como Carlos Chagas (1878-1934) e Arthur Neiva, e por campanhas sanitárias governamentais contra a febre amarela e a malária na capital federal ( Peryassú, 1908 , p.321-322). O numeroso material coletado durante a elaboração da tese teria sido depositado em Manguinhos, já consagrado centro de excelência no estudo da interseção doenças e seus insetos vetores ( Aragão, 1950 ; Benchimol, 1990 ).

## Coleções e estudos sobre mosquitos no Rio de Janeiro do início do século XX

Enquanto o Instituto de Manguinhos vinha se devotando ao estudo dos mosquitos e mantendo coleções desses insetos desde 1900 ( Fonseca Filho, 1974 ; Marchon-Silva et al., 1996 ),^1^ o Museu Nacional do Rio de Janeiro (MNRJ), dedicado ao estudo e à divulgação da história natural, tinha sob sua custódia vastas coleções institucionais de artrópodes desde meados do século XIX, porém compostas essencialmente de espécies de importância agrícola ( Lopes, 1997 ; Serejo, 2020 ). Os demais museus congêneres no mundo também eram dessa forma, até que as descobertas de Ronald Ross (1857-1932) e Charles Louis Alphonse Laveran (1845-1922) incriminando mosquitos como transmissores da malária mudassem esse cenário (Benchimol, Sá, 2006).

Assim, foram entomologistas agrícolas britânicos que verdadeiramente deram início à culicidologia, em particular à taxonomia e sistemática desses dípteros. Um deles, Frederick Vincent Theobald (1868-1930), admitido pelo Museu Britânico de História Natural (MBHN) para realizar estudos sobre mosquitos, necessitava de material, pois mesmo a coleção dessa tradicional instituição europeia tinha poucos exemplares de mosquitos. Era preciso colecionar em grande escala, examinar e comparar mosquitos de toda parte para, então, propor organizações taxonômicas e definir os táxons. Theobald e o MBHN criaram uma rede mundial de coletas de mosquitos, a partir de 1899, da qual Adolpho Lutz (1855-1940), do Instituto Bacteriológico de São Paulo, teria sido um dos mais eficientes e pioneiros cooperadores. O cônsul britânico no Pará, William Algernon Churchill (1865-1947), e o entomologista Carlos Moreira (1869-1946), do MNRJ, também enviaram mosquitos a Theobald em 1899 e 1900 (Belkin, Schick, Heinemann, 1971; Benchimol, Sá, 2006). Fenômeno semelhante se deu nos Estados Unidos, onde o Museu Nacional de História Natural, em Washington, procurou, rápida e competitivamente, estimular o colecionamento de mosquitos e capacitar entomologistas agrícolas em culicidólogos. Eles coletaram ou receberam volumoso material das Índias Ocidentais e das Américas do Norte e Central, organizando numerosa coleção.

Diferentemente dos museus de história natural de Londres e Washington, o MNRJ não parece ter estimulado os seus entomologistas agrícolas a se especializar no estudo dos mosquitos no raiar do século XX, ficando esse campo da entomologia, no Rio de Janeiro, restrito ao Instituto de Manguinhos. Por conta disso, o MNRJ não detinha uma coleção específica desses importantes insetos vetores quase duas décadas após ter início a supracitada revolução na culicidologia nos congêneres estabelecimentos britânico e norte-americano, até que, em abril de 1918, contratou Peryassú na função de naturalista (Lobo, 30 abr. 1918). O contrato foi renovado em 1920, terminando em 31 de dezembro de 1922 ( Brasil, 1921 , p.544).

## A Coleção Peryassú

Nos cinco anos de atuação no MNRJ (1918-1922), Peryassú organizou uma numerosa e diversa coleção específica de mosquitos, posteriormente nomeada Coleção Peryassú. Esta “coleção histórica” foi incorporada à Coleção de Diptera do museu, instituída pelo entomologista especialista em moscas Dalcy de Oliveira Albuquerque (1918-1982), a partir da década de 1940 ( Carvalho et al., 2002 ).

Um inventário da Coleção Peryassú, confeccionado entre 2011 e 2018, revelou que o acervo remanescente no MNRJ continha 722 mosquitos de 62 espécies, de 18 gêneros e 24 subgêneros de Culicidae, incluindo-se os tipos da espécie *Cellia oswaldoi* (= *Anopheles oswaldoi* ) descrita por Peryassú, em 1922 (Silva-do-Nascimento, Motta, Lourenço-de-Oliveira, 2020). Os mosquitos procederam majoritariamente do então Distrito Federal (cidade do Rio de Janeiro) e do estado do Rio de Janeiro, como revelam o referido inventário e relatório das atividades do museu ( Brasil, 1920 , p.39). A coleção continha mosquitos de localidades fluminenses como Xerém, Magé, Petrópolis, Porto das Caixas, Itatiaia e Angra dos Reis, e dos estados de Pernambuco, Pará, Bahia, Minas Gerais, Espírito Santo, São Paulo, Paraná, Santa Catarina e Rio Grande do Sul. Todo o material foi coletado no Brasil, exceto por um grupo de 19 exemplares de dez espécies capturados no Japão, entre 1915 e 1919. Como veremos a seguir, tais exemplares exóticos à fauna brasileira referem-se à permuta de mosquitos identificados pelo culicidologista japonês Shinichiro Yamada (1883-1937), que descreveu duas dezenas de espécies novas de mosquitos, entre 1917 e 1932 (Kurihara, Kurahashi, Shinohara, 2001).

**Figura 2:**
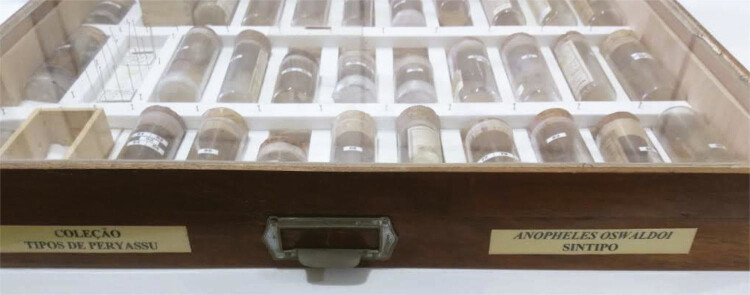
Gaveta da Coleção Peryassú, Departamento de Entomologia, MNRJ (Fotografia de R. Lourenço-de-Oliveira, agosto de 2018)

Os mosquitos japoneses depositados na Coleção Peryassú foram montados como os demais das coleções organizadas por Yamada ainda existentes (Kurihara, Kurahashi, Shinohara, 2001), empregando metodologia distinta da usada por Peryassú no MNRJ.^2^ Em sua coleção, Peryassú adotou o método de montagem que os culicidologistas pioneiros recomendavam para a melhor preservação de mosquitos (Benchimol, Sá, 2006, p.148): tubos cilíndricos de vidro com tampa de cortiça, na qual os alfinetes portando os insetos eram espetados ( Figura 2 ).

O inventário da Coleção Peryassú encontrou 329 tubos, dos quais 317 continham de um a 12 mosquitos, que poderiam estar presos individualmente ou agrupados em um único alfinete; não foram colocadas etiquetas nos alfinetes. Doze tubos estavam vazios, e os espécimes que haviam sido ali montados desapareceram (Silva-do-Nascimento, Motta, Lourenço-de-Oliveira, 2020, p.13, 20, 23). Por outro lado, muitos espécimes se achavam fora de seus tubos originais, montados em alfinetes como os demais, porém fixados em placa de isopor dentro de caixas de feitura recente, ao lado das quais, na maioria das vezes, estavam os respectivos tubos vazios. Os tubos originais de alguns espécimes, porém, não foram encontrados (p.13, 20, 23).

Não foi encontrado livro de tombo da Coleção Peryassú. Toda informação sobre os exemplares armazenados em cada tubo se achava no rótulo de papel, padronizado pelo MNRJ, colado ao tubo, no qual Peryassú anotou, à mão, com nanquim, o nome da espécie e de seu autor, o número da espécie e do exemplar e as notas sobre o local de coleta, muitas vezes enriquecidas com o nome do coletor e com os dados sobre a biologia ou a importância sanitária da espécie.

Peryassú não coletou todo o material brasileiro depositado em sua coleção, e nem sabemos dimensionar que parte lhe teria sido transferida por guardas de serviços de combate a vetores de doenças como febre amarela e malária ou técnicos. Cabe lembrar que, durante a organização da coleção de mosquitos no MNRJ, Peryassú também atuou como inspetor do Serviço de Profilaxia Rural, no Rio Janeiro (1920-1922). Entre as atividades desses serviços sanitários, tanto rotineiramente quanto em ações especiais, incluía-se a captura de mosquitos. Por exemplo, após excursão para o diagnóstico das condições sanitárias do Vale do Rio Doce, no Espírito Santo, em 1922, Peryassú indicou que deveriam ser “capturados, com tubos de vidro ou saco de filó, todas as manhãs, entre 6 e 7 horas, os mosquitos que, por acaso tenham entrado no barracão” (O Paiz, 30 abr. 1923, p.3). Assim, é muito provável que a maior parte dos mosquitos depositados na Coleção Peryassú tenha sido coletada por auxiliares.^3^ Talvez por isso, o rótulo da maioria dos tubos não informava o nome do coletor.

Até mesmo ex-companheiros de Manguinhos coletavam mosquitos para Peryassú no contexto dessa empreitada no MNRJ. Lauro Travassos (1890-1970), helmintologista e entomologista, forneceu a Peryassú mosquitos por ele coletados em julho e novembro de 1918, em Angra dos Reis [ *Culex imitator, Janthinosoma cingulata* (= *Psorophora cingulata* ), *Cellia tarsimaculata* (= *Anopheles aquasalis* ), *Manguinhosia lutzii* (= *Anopheles lutzii* ), *Arribalzagia maculipes* (= *Anopheles maculipes* ) e *Cycloleppteron intermedium* (= *Anopheles medialis* )], e no *campus* de Manguinhos ( *Psorophora ciliata* ), que o naturalista depositou na sua coleção. Esse detalhe revela que Peryassú ainda mantinha intercâmbio científico com os profissionais de Manguinhos. Houve também contribuições menos diversas ou numerosas que a de Travassos, como a do entomologista Dario Mendes (1892-1963), que doou mosquitos de quatro espécies do Recife (1919) e Belém (1920-1921), dos parasitologistas Aristides Marques da Cunha (1887-1949) e Manoel Pirajá da Silva (1873-1961) e do comandante J. Cordeiro, que doaram, cada qual, uma espécie capturada, respectivamente, no Rio Grande do Sul (1918), na Bahia (1919) e no rio Madeira (Amazonas?) (1919).

Digna de nota foi a doação de um exemplar de mosquito silvestre, *Wyeomyia lunata* (= *Isostomyia lunata* ), capturado em Itatiaia por Carlos Moreira, em 1903, 15 anos antes de Peryassú dar início à coleção. Interessante o fato de Moreira ter participado da primeira rede de coletas e remessas de mosquitos ao MBHN, em 1899 (Belkin, Schick, Heinemann, 1971; Benchimol, Sá, 2006), e ter mantido o trabalho de coleta e de guarda de mosquitos desde 1903, sem ter constituído uma coleção específica desse grupo de dípteros no MNRJ ( Pamplona et al., 2000 ; Carvalho et al., 2002 ).

Os depósitos de mosquitos brasileiros na Coleção Peryassú foram realizados de maneira heterogênea entre 1918 e 1922. São dos primeiro e último anos de atividade no museu os maiores registros de entradas de material, correspondendo a cerca de 38% e 31% dos tubos. Houve grande desaceleração de depósitos em 1919 e 1920, sendo que neste último ano se agregou o menor número de tubos, somente 18, correspondendo a 3% das entradas.

Excluindo-se a supracitada *Wyeomyia lunata* , de 1903, o espécimen mais antigo na coleção refere-se a um macho de *Stegomyia calopus* (= *Aedes aegypti* ) capturado na cidade do Rio de Janeiro em 8 de agosto de 1912, seis anos antes do início do contrato de Peryassú no MNRJ (Silva-do-Nascimento, Motta, Lourenço-de-Oliveira, 2020, p.3). Cronologicamente, após esses dois espécimes, a próxima entrada foi de um *Trichoprosopon compressum* , de origem desconhecida, coletado em 14 de janeiro de 1918, mais de três meses antes do contrato de Peryassú. A Coleção Peryassú, portanto, não foi iniciada com depósitos de mosquitos coletados somente após o início do trabalho dele no museu, em abril de 1918; Peryassú adicionou à coleção material que ele ou outros já tinham em seu poder.

Os dois últimos depósitos na coleção corresponderam a dois espécimes de *Protoculex serratus* ( *= Aedes serratus* ) e de *Teaniorhynchus chrysonotum* (= *Coquillettidia chrysonotun* ), capturados em 28 de dezembro de 1922, três dias antes de findar as atividades de Peryassú no MNRJ (Silva-do-Nascimento, Motta, Lourenço-de-Oliveira, 2020, p.10, 38).

O número de exemplares por espécie depositado foi grande. As espécies com maior quantidade foram: *Stegomyia calopus* (125 em 32 tubos), *Culex scapularis* (= *Aedes scapularis* ) (111 em 37 tubos), *Culex fatigans* (= *Culex quinquefasciatus* ) (89 em 29 tubos), *Taeniorhynchus chrysonotum* (47 em 18 tubos), *Protoculex serratus* (42 em 20 tubos), *Taeniorhynchus titillans* (= *Mansonia titillans* ) (34 em 21 tubos), *Culex coronator* (30 em 11 tubos) e *Psorophora ciliata* (20 em 16 tubos). Vinte e uma espécies, 11 do Brasil e dez do Japão, apresentam apenas uma entrada na coleção.

## Doações e permuta: intercâmbio científico e militância pelo ensino da entomologia médica e sanitarismo

O número de exemplares de mosquitos reunidos na Coleção Peryassú era seguramente bem maior do que o contabilizado no inventario feito entre 2011 e 2018. O fato de vários tubos terem sido encontrados vazios constitui por si só um indicador de que a coleção era mais numerosa. Mas os principais indícios são as extrações de lotes dessa coleção com a finalidade expressa de doação e/ou permuta de mosquitos, realizadas por Peryassú no período em que esteve no museu, à época vinculado ao Ministério da Agricultura, Indústria e Comércio. O relatório das atividades de 1919 remetido ao presidente da República assim reportou a atuação do naturalista contratado sobre as doações:

Pelo professor Antonio Peryassú, que realizou várias excursões no estado do Rio, foram coligidos muitos dípteros para a coleção do Museu e organizadas 13 coleções de culicídeos destinados a diferentes institutos científicos do estrangeiro e estabelecimentos de ensino no Brasil (Brasil, 1920, p.39).

Tanto os relatórios dos feitos anuais do MNRJ quanto a imprensa leiga e acadêmica da época reportaram o preparo de numerosas amostras de mosquitos para doação a instituições de pesquisa, controle de endemias e, sobretudo, de ensino, destacando-se as faculdades de medicina, no Brasil e no exterior. Algumas doações ganharam destaque na imprensa por terem acontecido no contexto de relações e visitas diplomáticas, em cuja comitiva havia responsáveis por setores de saúde pública e pesquisadores da biomedicina. No que diz respeito a faculdades de medicina no estrangeiro, existem registros de doações para aquelas de Buenos Aires, em 1921, de Praga e de Assunção. O lote destinado ao Paraguai foi levado, em mãos, em 1920, por Edgard Roquette-Pinto (1884-1954), que regeria a cadeira de fisiologia na escola paraguaia. Os exemplares de mosquitos doados a Praga, em 1921, tiveram o próprio embaixador tcheco, Jan Havlasa (1883-1964), como intermediário (Correio da Manhã, 16 abr. 1920; O Brasil Médico, 28 maio 1921; O Paiz, 28 ago. 1921).

Com o intuito de permuta de material para pesquisa, o MNRJ também enviou um lote de 27 mosquitos brasileiros de 23 espécies preparado por Peryassú ao Instituto Kitasato, de Tóquio, como desdobramentos da visita de intercâmbio científico de Mikinosuke Miyajima (1872-1944), pesquisador do instituto japonês, ao museu, em 1919 (O Imparcial, 17 abr. 1919; Lobo, 22 abr. 1919). Em novembro de 1920, o cirurgião-chefe de um dos navios da esquadra japonesa em passagem pelo Brasil, Bongero Abe (1881-1955), foi o portador de uma coleção de mosquitos doados pelo Instituto Kitasato ao MNRJ, que incluía “entre eles diferentes espécimes do *Culex* japonezes ( *sic* ), bem como *Anopheles sinensis* ” (Correio Paulistano, 30 nov. 1920, p.1). Esse detalhe indica se tratar da amostra de 19 mosquitos preparada por Yamada e incorporada à Coleção Peryassú.^4^ Uma amostra complementar de mosquitos brasileiros teria sido organizada por Peryassú e enviada, subsequentemente, ao Instituto Kitasato, como retribuição.

No Brasil, por sua vez, as doações de lotes de mosquitos foram feitas às faculdades de medicina do Rio de Janeiro, do Pará e de Belo Horizonte, em 1919. Outras instituições da capital mineira também receberam mosquitos estudados por Peryassú: a filial do Instituto Oswaldo Cruz, atual Fundação Ezequiel Dias, e a Repartição de Higiene, equivalente a uma secretaria de saúde na época (Lobo, 26 set. 1919; O Imparcial, 28 set. 1919; Estado do Pará, 30 nov. 1919). Sobre a oferta de material entomológico para a faculdade carioca, noticiou *O Paiz* (8 abr. 1919, p.5):

O Museu Nacional enviou à Faculdade de Medicina do Rio de Janeiro uma coleção de 21 espécies de culicídeos, preparada e classificada pelo naturalista dr. Antonio Peryassú, e uma coleção de insetos organizada pelo praticante Dario Mendes, do Laboratório de Entomologia Geral e Aplicada, destinadas ambas ao Laboratório de História Natural daquela Faculdade.

Muito além da lógica da coleção zoológica e de sua finalidade na taxonomia, Peryassú usou as notas dos rótulos dos tubos da coleção para aquela que o norteou desde a sua tese em 1908: o estudo dos mosquitos a serviço da vigilância e do controle das doenças por eles transmitidas e em prol do sanitarismo. Diferentes setores do museu preparavam coleções para doação com finalidade didática (MNRJ, 1920, p.49), e Peryassú participava dessa iniciativa, especialmente ao adotar a estratégia de adicionar dados usualmente não incluídos nas coleções zoológicas, tais como: informações sobre a distribuição geográfica, comportamento, *habitat* larvário, tempo de desenvolvimento pupal e, sobretudo, o papel da espécie como vetor comprovado ou potencial de doenças nos rótulos dos tubos, inclusive naqueles doados.^5^

Talvez pela descontinuidade no estudo de mosquitos no MNRJ, após o encerramento das atividades de Peryassú, em 1922,^6^ a coleção tenha sido por muitos desconhecida. O exame de seus exemplares por especialistas e referências à Coleção Peryassú aparecem quase cinquenta anos após o último depósito de mosquitos. Assim, a coleção foi examinada, em 1969, pelo taxonomista John Belkin (1913-1980), que fez um breve relato da dificuldade em localizar, ali, material-tipo de espécies descritas por Peryassú (Belkin, Schick, Heinemann, 1971, p.16, 40).

Em 1987, o exame dos espécimes etiquetados por Peryassú como *Cellia allopha* (= *An. allopha, nomen dubium* ) confirmaram que o autor definira a sua espécie a partir da mistura de espécimes de distintas espécies, conforme previamente indicado por Lourenço-de-Oliveira e Deane (1984) . Em 1997, Flores Mendoza (1999) dissecou a genitália masculina de um dos síntipos de *Cellia oswaldoi* , e Motoki et al. (2007) redescreveram a espécie, elegendo um lectótipo. Fora do contexto dos estudos de taxonomia de mosquitos, a pouco conhecida Coleção Peryassú, dita de “valor histórico”, é citada na literatura muito esporadicamente já no século XXI ( Carvalho et al., 2002 ; Fernandes, Rodrigues Junior, Couri, 2012; Messias et al., 2012 ). Finalmente, o inventário da Coleção Peryassú foi disponibilizado em 2020 (Silva-do-Nascimento, Motta, Lourenço-de-Oliveira, 2020).

## Um lote de mosquitos como testemunho

No dia 2 de setembro de 2018, a Coleção Peryassú e todo o restante do acervo entomológico estimado em mais de 12 milhões de espécimes que se encontravam no edifício central do museu, Palácio da Quinta da Boa Vista, foram destruídos por um incêndio de grande proporção (Sá, Sá, Lima, 2018; Serejo, 2020 ). Os únicos registros do que teria sido a Coleção Peryassú passaram a ser o inventário preparado até a antevéspera do incêndio (Silva-do-Nascimento, Motta, Lourenço-de-Oliveira, 2020) e algumas fotografias digitais, como a de uma gaveta da coleção ( Figura 2 ) e a da genitália masculina do lectótipo de *Cellia oswaldoi* , disponível em Flores Mendoza (1999 , p.80).

Como o preparo de amostras de mosquitos por Peryassú visando à doação ou à permuta para instituições de pesquisa e ensino no Brasil e exterior foi considerável, supomos que alguns espécimes extraídos da coleção original ainda estivessem disponíveis nos locais para onde foram doados. A busca realizada junto a museus e instituições de ensino e pesquisa recebedores das doações feitas por Peryassú, segundo relatórios do MNRJ e notas publicadas nos jornais da época, não encontrou nenhum espécime.

**Figura 3:**
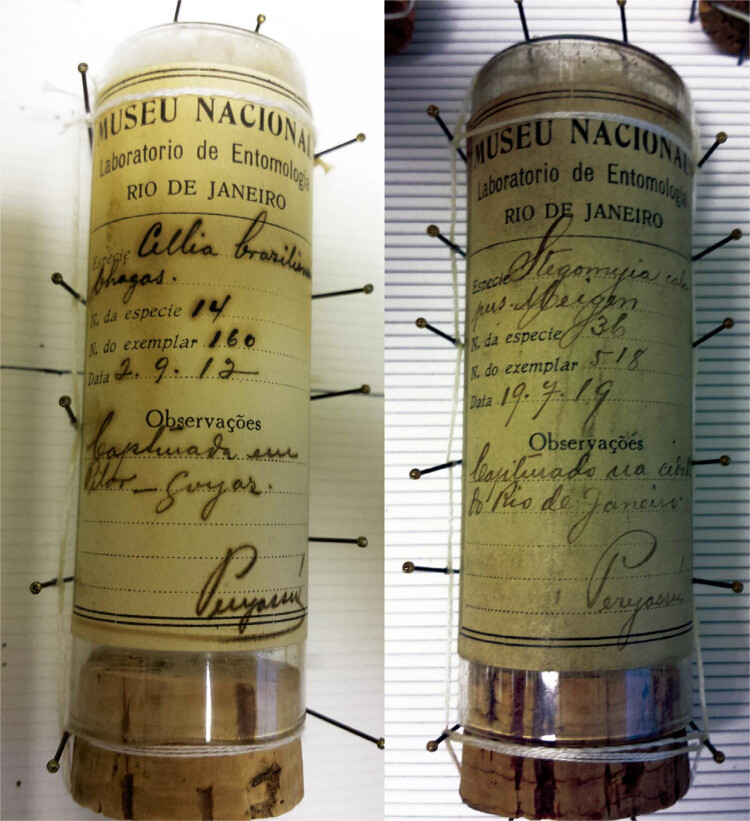
Tubos da Coleção Peryassú preservados na Coleção de Artrópodes do Instituto Pasteur (Fotografia de R. Lourenço-de-Oliveira, outubro de 2017)

**Figura 4:**
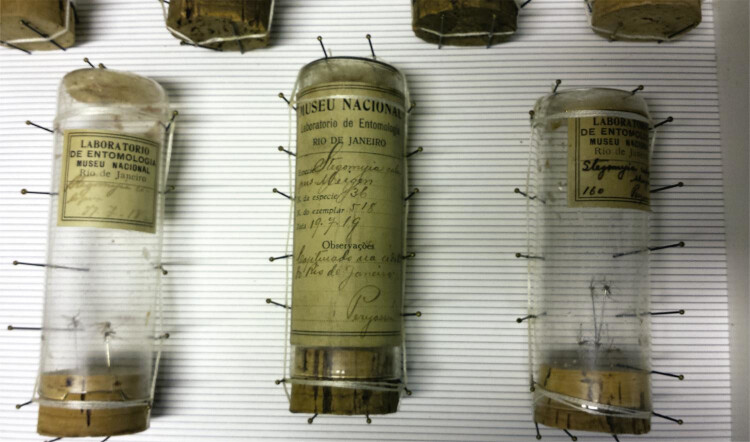
Grupo de tubos da Coleção Peryassú organizado em uma caixa na Coleção de Artrópodes do Instituto Pasteur, onde se observa a existência de mais de um mosquito por tubo, estratégia que Peryassú utilizou frequentemente no preparo de sua coleção no MNRJ (Fotografia de R. Lourenço-de-Oliveira, outubro de 2017)

Entretanto, amostras da Coleção Peryassú foram por nós localizadas no Instituto Pasteur, de Paris, em 2017. Tal lote de mosquitos fora doado à Faculdade de Medicina de Paris, de cujo Laboratório de Parasitologia foi transferido para a Coleção de Artrópodes do Instituto Pasteur, em 1980, pelo médico e entomologista François Rodhain (1939- ), e desde então está mantido no Pavilhão Nicolle, sob a guarda da Unité Arbovirus et Insectes Vecteurs (Rodhain, Boutonnier, 1984, p.271). A doação foi provavelmente feita, em mãos e em parcelas, a Émile Brumpt (1877-1951), professor responsável pela cátedra de parasitologia da escola parisiense desde 1919, que visitou o Rio de Janeiro e o MNRJ em 1922, 1924 e 1927, e interagiu estreitamente com os diretores Bruno Lobo (1884-1945), Arthur Neiva e Roquette-Pinto (Brumpt, 1922-1924; Le professeur..., 19 maio 1927). O aspecto físico desse material, ou seja, os tubos e rótulos do MNRJ, quase sempre assinados por Peryassú ( Figura 3 ), é idêntico ao destruído pelo incêndio, confirmando se tratar de doação de lote da Coleção Peryassú.^7^ Parte dos exemplares está em 24 tubos contendo um ou mais mosquitos, organizados em cinco caixas de papelão ( Figura 4 ). Outros 36 exemplares estão montados separadamente em alfinetes, dez deles ainda associados a etiquetas originais do MNRJ. O lote apresenta uma diversidade interessante de espécies de diferentes biomas brasileiros: são 24 espécies de 13 gêneros segundo a nomenclatura da época e que, hoje, corresponde a sete gêneros. São procedentes de Goiás, Pará, Minas Gerais, Espírito Santo, Paraná e Rio de Janeiro, este último o mais representado com exemplares de Tijuca, Águas Férreas (Laranjeiras), São Cristóvão, Santa Cruz ou outros bairros não especificados, da capital federal, mas também de Niterói, Xerém, Magé e Petrópolis.^8^ A espécie mais representada, certamente por sua importância sanitária, é *Stegomyia callopus* . Todos os mosquitos foram coletados entre 1918 e 1922, com uma única e intrigante exceção: uma *Cellia braziliensis* (= *Anopheles braziliensis* ), coletada em Pilar, Goiás, no dia 2 de setembro de 1912, seis anos antes de Peryassú ingressar no MNRJ. Interessante ainda é o fato de uma espécie de mosquito, *Gualteria oswaldi* (= *Aedes serratus* ), capturada em Goiás, não estar representada na Coleção Peryassú que permaneceu na instituição (Silva-do-Nascimento, Motta, Lourenço-de-Oliveira, 2020), mas que compõe o lote do Instituto Pasteur.

## Considerações finais

As coleções biológicas, nelas incluídas as de insetos, são cada vez mais consideradas importantes patrimônios institucionais e nacionais (Costa et al., abr.-jun. 2008; Rangel, 2009 ). A inclusão formal de um espécime qualquer em um acervo institucional confere a ele caráter precioso, único ( Pomian, 1985 , p.53). Caráter ainda mais precioso adquire um espécime-tipo ou mesmo espécies coletadas em locais de onde foram extintas, como vários depositados na Coleção Peryassú.

Com o incêndio, perdeu-se a quase totalidade do que fora esse patrimônio, exceto pelo lote de mosquitos conservado no instituto francês até recentemente desconhecido, que consiste no único testemunho físico de como foi um dia a Coleção Peryassú antes de sua total destruição, em 2018.

Graças à existência desse lote e do inventário da Coleção Peryassú podemos ter indícios diretos ou indiretos da biodiversidade de mosquitos das localidades investigadas na época, das atividades de pesquisa e ensino e das ações de doação e permuta empreendidas por Peryassú, assim como do seu entendimento sobre o papel de algumas espécies de mosquitos como vetores de doenças. Portanto, a produção, o arquivamento e a divulgação digital de inventários, de catálogos detalhados e dos registros de doação e permuta de material e a geração de dossiês de imagens dos espécimes de coleções biológicas devem ser prática quotidiana nos museus e institutos de pesquisa.

## NOTAS

1 O Instituto de Manguinhos valorizou suas coleções ao publicar um catálogo com primorosa programação visual *art nouveau* , listando 95 espécies de mosquitos ali colecionados (IOC, 1909).

2 Os mosquitos das coleções de Yamada atualmente existentes no Museu Nacional de Ciência e no Instituto Nacional de Doenças Infecciosas, de Tóquio, estão montados em pequenas caixas de papelão medindo 45x60x45mm, com tampa de vidro, tal como os encontrados na Coleção Peryassú.

3 Forte indicativo de que Peryassú não coletou a maioria dos mosquitos da sua coleção no MNRJ é o fato de nela só estar depositado um único espécime coletado durante a sua estada no Vale do Rio Doce, em março de 1922: *Wyeomyia lunata* . Por outro lado, 38 espécimes foram lá coletados seis a sete meses após o seu retorno ao Rio de Janeiro.

4 Dois exemplares de *Anopheles sinensis* , de 5 de julho de 1919, identificados por Yamada, foram encontrados na Coleção Peryassú. Havia também espécimes de *Culex* procedentes do Japão no lote identificado por Yamada, como *Culex tritaeniorhynchus, Culex mimeticus* e *Culex pipiens* , que provavelmente correspondem aos referidos *Culex* da nota do *Correio Paulistano* .

5 Quatro exemplos de conteúdo de rótulo são: “Espécie doméstica, vetora da filariose e febre amarela. Capturado no Museu Nacional” (n. 5.141, *Stegomyia calopus* ); “Espécie espalhada no Brasil, vetora da malária” (n.13.014, *Cellia tarsimaculata* ); “Espécie doméstica e noturna, vetora da filariose e provavelmente da lepra” (n.13.215, *Culex fatigans* ); “Criado a partir de larva coletada em 20 de junho de 1919 e transformada em ninfa em 18 de outubro de 1919, a qual eclodiu em 20 de outubro de 1919. Espécie silvestre, diurna” (n.922, *Limatus durhamii* ).

6 A exceção foi um conjunto de ações, especialmente de coleta e organização de acervo, realizadas particularmente na segunda metade dos anos 1990, em torno de uma coleção de mosquitos doada por Ivan Ricciardi, durante atuação como pesquisador visitante no MNRJ nessa década. Era composta essencialmente por anofelinos e organizada durante atividades de Ricciardi e outros no Serviço Nacional de Malária, no Instituto de Malariologia e na Fundação Rockefeller, principalmente nos anos 1950-1960. (Souza, Khouri, Lopes, 1994, p.187; Khouri, 1995 , p.34; Ricciardi, Khouri, Lopes, 1997 ).

7 Acreditamos que foi doação à Faculdade de Medicina francesa, e não permuta, porque o inventário da Coleção Peryassú não encontrou espécimes do estrangeiro, exceto os identificados por Yamada. Mesmo assim, não houve permuta entre Peryassú e Yamada, segundo o resultado de busca feita em coleções onde estão suas amostras, a maioria no Museu Nacional de Ciências, de Tóquio.

8 O lote do Instituto Pasteur contém as seguintes espécies: *Stegomyia callopus* (7 tubos + 1 alfinetado separadamente), *Arribalzagia pseudomaculipes* ( *= Anopheles pseudomaculipes* ) (1 tubo + 3 alfinetados), *Cellia brasiliensis* (1 tubo + 2 alfinetados), *Cellia argyritarsis* (= *Anopheles argyritarsis* ) (1 tubo + 6 alfinetados), *Culex quinquefasciatus* (1 tubo + 3 alfinetados), *Culex fatigans* (2 tubos), *Culex apicalis* (= *Psorophora cingulata* ) (2 tubos cada), *Bancroftia albicosta* (= *Orthopodomyia albicosta* ), *Cellia allopha, Culex confirmatus* (= *Aedes scapularis* ), *Carrollia iridescens* (= *Culex iridescens* ), *Chrysoconops fulvus* (= *Aedes fulvus* ), *Culicelsa taeniorhynchus* (= *Aedes taeniorhynchus* ), *Janthinosoma discrucians* (= *Psorophora discrucians* ), *Gualteria oswaldi, Limatus durhamii* (1 tubo cada), *Anopheles albimanus* (provavelmente *Anopleles aquasalis* ) (7 alfinetados), *Cellia tarsimaculata* ( *= Anopheles aquasalis* ) (4 alfinetados), *Taeniorhynchus chrysonotum, Culex coronator* (3 alfinetados cada), *Taeniorhynchus albicosta* (= *Coquillettidia albicosta* ), *Psorophora posticata* (= *Psorophora ferox* ), *Psorophora ciliata* e *Anopheles tarsimaculatus var. oswaldoi* ( *Anopheles oswaldoi* ) (1 alfinetado cada).
